# A qualitative study on patients’ and health care professionals’ perspectives regarding care delivered during CIED operation

**DOI:** 10.1186/s12913-024-10546-7

**Published:** 2024-01-15

**Authors:** Min Zhou, Huilin Zhou, Xiong Zhang, Xiaorong Jin, Xu Su, Yangjuan Bai, Wei Wei, Yimei Zhang, Fang Ma

**Affiliations:** 1 Department of Nursing, The First Affiliated Hospital of Kunming Medical University, No. 295, Xichang Road, 650032 Kunming, China; 2 School of Nursing, Kunming Medical University, Kunming, China; 3 Cardiology Department, The First Affiliated Hospital of Kunming Medical University, Kunming, China; 4 Digestive Surgery Department, The First Affiliated Hospital of Kunming Medical University, Kunming, China

**Keywords:** Qualitative study, Cardiac implantable electronic devices, Intraoperative care

## Abstract

**Background:**

Cardiac implantable electronic devices (CIEDs) has proven to be an invaluable tool in the practice of cardiology. Patients who have undergone CIED surgery with local anesthesia may result in fear, insecurity and suffering. Some studies have put efforts on ways to improve intraoperative experience of patients with local anesthesia, but researches concerning experiences of CIED patients during surgery is in its infancy.

**Methods:**

Based on semi-structured and in-depth interviews, a qualitative design was conducted in a tertiary general hospital in China from May 2022 to July 2023.Purposeful sampling of 17 patients received CIED surgery and 20 medical staff were interviewed. Thematic analysis with an inductive approach was used to identify dominant themes.

**Results:**

Four themes emerged from the data: (1) Safety and success is priority; (2) Humanistic Caring is a must yet be lacking; (3) Paradox of surgery information given; (4) Ways to improve surgery experiences in the operation.

**Conclusions:**

Intraoperative care is significant for CIED surgery. To improve care experience during surgery, healthcare professionals should pay attention to patients’ safety and the factors that affecting humanistic caring in clinical practice. In addition, information support should consider information-seeking styles and personal needs. Besides, the four approaches presented in this study are effective to improve the intraoperative care experience.

**Supplementary Information:**

The online version contains supplementary material available at 10.1186/s12913-024-10546-7.

## Background

Cardiac implantable electronic devices(CIEDs) is a term that encompasses a number of devices that provide a treatment of bradyarrhythmias, ventricular tachyarrhythmias, and advanced systolic heart failure [1, 2]. It has proven to be an invaluable tool in the practice of cardiology, and implantation rates continue to rise, with more than 600,000 CIEDs practiced each year [3]. The CIEDs include implantable cardioverter defibrillators (ICD) and cardiac resynchronization therapy (CRT) devices [4]. Most CIEDs typically involve local anesthesia, and are placed under the skin in the left shoulder region, with leads connecting to the vasculature of the heart [5, 6]. Although some centers have used local anesthesia with sedation for CIEDs implantation, there is still debate regarding the safety of using sedation because of possible undesirable side effects, such as hypoxaemia, hypotension, nausea and vomiting. Furthermore, more elderly patients (mean age ≥ 70 years) with many medical comorbidities and people with advanced heart disease receive this procedure, which might lead to high sedation induced risk [7, 8]. Hence, in most cases of CIEDs procedure, patients will remain awake without sedation, which may result in patients’ fear, insecurity and suffering for their vulnerability and sense of losing control in the operation [9–11].

Previous researches have shown that the conscious state of patients receiving CIEDs with local anesthesia may lead to many adverse effects. In a study by Selwyn et al [12] indicated that CIED patients with local anesthesia experience severe pain that may be of a long duration. Anne et al [13] showed that a considerable number of patients receiving an ICD had symptoms of depression and anxiety pre-ICD implantation, and these symptoms level would increase during the operation. In another study, it was also stressed that an important minority of CIED patients reported severe pain during the procedure, suggesting that peri-operative pain management in CIED procedures warrants attention [14]. Moreover, in Chinese culture, the heart is regarded as the home of the emotions, cognition and even the soul. Therefore, receiving a diagnosis of heart disease signals a life-threatening illness. When they have to receive the cardiac surgery, Chinese patients may become particularly scared and anxious when facing the operation [15, 16]. All above suggest that undergoing CIED operation signals an enormous challenge and pressure for patients.

Some researches have put efforts on ways to improve the care of patients with local anesthesia during operation [17]. Studies have shown that non-pharmacological treatments, such as preoperative education, massage therapy etc. are effective for patients with local anesthesia to improve their psychological well-being during surgery, and they are relatively risk-free as well [7, 18]. Haugen et al [19] indicated that intraoperative communication between health care professionals(HCPs) and patients decreased their anxiety level and met patients’ needs under the orthopedics surgery [20]. Bergman et al [21] noted that during the spinal anesthesia, the presence of various technical equipment and devices raised patients’ concern, and seeing HCPs being with them made patients feel safe and calm down. Another study by Moon et al [22] demonstrated that it is important for patients with local anesthesia to feel nearness during surgery through contact, such as holding their hand, which would reduce patients’ anxiety. Moreover, Merakou et al [23] demonstrated that meditation music could reduce patients’ stress and kept them calm during cataract surgery. In recent years, with the development of technology, virtual reality has also been used to reduce pain and negative emotions of patients with local anesthesia during the operation [24]. However, the intraoperative care of patients with local anesthesia is unstandardised and largely determined by HCPs’ preference, with patients preference and needs being ignored [25]. Besides, most researches in intraoperative care of CIED patients has centred on technology and its application, rather than on patient experience, and study concerning intraoperative experiences for CIED patients is in its infancy [14, 26]. Nowadays, it is advacated that interventions based on stakeholders’ perception can improve health outcomes. Therefore, it is imperative to explore patients’ and HCPs’ perceptions about their experiences of CIED implantation under local anesthesia context, which can contribute to developing patient-centered care intervention program. Hence, based on patients’ and HCPs’ perspectives, this study aims to explore and analyse their intraoperative care experiences, including their feelings, attitudes, perceptions and some approaches to improve the experience of surgery.

## Method

### Study design

This study was conducted using a descriptive phenomenology qualitative design based on semi-structured and in-depth interviews. Qualitative methodological approaches are appropriate when the research seeks to describe the essence of a phenomenon by exploring it from the perspective of those who have experienced it [27, 28]. The goal of phenomenology is to describe the meaning of this experience, both in terms of what was experienced and how it was experienced in nursing science [28, 29]. In this study, the research question is “What are the care experience of patients and HCPs during CIED surgery”. The consolidated criteria for reporting qualitative research (COREQ) checklist was used [30].

### Setting, participants and sampling method

The study was conducted in Yunnan province for its historical, geographical and cultural characteristics, which may result in unique aspect that will contribute our understanding of the study issue. Firstly, Yunnan province has historically been a strategic location on the ancient southern Silk Road and shares borders with Myanmar, Lao, and Vietnam [31]. Since 2013, the Belt and Road Initiative has been implemented in China, with ASEAN Free Trade Area mainly being implemented through Yunnan, which stress not only the goods, but also Chinese special culture [32]. Secondly, compared to the coastal areas, such as Shanghai, Zhejiang, and Guangdong, then on-coastal Yunnan is more traditional and may present Chinese cultural characteristics [33]. Thirdly, many ethnic minority groups live in Yunnan province, the existence of the many minority groups makes Yunnan a good base for cultural studies [32]. All above suggest that choosing Yunnan province as the study set may be a major attraction for academic research and provide cultural diversities concerning our study topic.

Based on purposeful sampling, this study recruited CIED patients and HCPs including physicians and nurses from a tertiary general hospital in Yunnan Province. The patients met the following inclusion criteria were sought:(a) age ≥ 18; (b) Undergoing first successful CIED surgery;(c) no mental illness and well recovered after operation. The inclusion criteria for HCPs were that they have participated in CIED surgery. Having obtained the participants’ informed consent, the investigator established rapport and a mutually convenient interview time with the participants was scheduled.

### Data collection

Semi-structured, face-to-face interviews were conducted with participants between May 2022 and July 2023. A semi-structured interview guide, constructed by the authors, was revised based on respondents’ feedback as the interviews progressed. The interview guide cited from supplementary file. The participants chose the time of interview and its’ location at meeting room of the department, which offered a quiet environment. A pilot study was conducted with two patients and two HCPs before the formal interview.

Interviews were conducted by the first author who has undergone systematic qualitative research training. Participants were encouraged to talk widely and freely about their experiences and perspectives during CIED surgery.

The interviews were audio-recorded and transcribed verbatim with duration ranging from 15 to 40 min. The interviews continued until the data was saturated when no new concepts emerged. The recording was transcribed within 24 h after each interview. In addition, a copy of the interview transcript was sent to each participant for verification.

### Data analysis

Qualitative thematic analysis with an inductive approach was used to identify dominant themes relating to the participants’ perspectives and experiences during CIED operation. Based on Braun and Clarke, the thematic analysis was carried out in six stages [34]: (1) Each transcript was read by two researchers, who listened to the audio recording carefully multiple times in order to get a sense of the whole; (2) The researchers identified initial codes inductively using the NVivo 11.0 software; (3) From the initial codes, themes that represented the phenomenon under study were constructed; (4) Two other researchers reviewed and validated the constructed themes for thematic validity and reliability; (5) Themes were named and defined; (6) finally, the final synthesis of the results were constructed and confirmed through review by all authors.

### Ethical considerations

The study was conducted in accordance with the Helsinki Declaration and was approved by the ethics committee of the hospital. Written informed consent was obtained from each participant. The content of interviews would be confidential, anonymous and used solely for this research.

## Results

### Participants characteristics

A total of 18 CIED patients were interviewed including 11 males and seven females, aged between 19 and 88 years. 20 HCPs took part in this study, including 13 physicians and seven nurses. Participants were numbered in turn by using the quotes, the quotes were as follows: patient(P),healthcare professional(HCP). The participants’ characteristics are summarized in Tables 1 and 2.

**Table 1:** Demographic characteristics of study participants (*N* = 18)

ID	Gender	Age	Marital Status	Education	Nation	Diagnosis
P1	Male	54	married	Primary school	Han	Chest Distress
P2	Male	58	married	Senior high school	Yi	Chest Distress
P3	Male	20	single	Senior high school	Han	Arrhythmia
P4	Male	56	married	Senior high school	Han	Bradycardia
P5	Female	78	married	Junior high school	Han	Fibrillation
P6	Female	55	married	Junior high school	Han	DilatedCardiomyopathy(DCM)
P7	Male	48	married	Primary school	Han	DCM
P8	Female	75	married	Primary school	Han	CardiacInsufficiency
P9	Female	65	married	Primary school	Han	Bradycardia
P10	Male	64	married	Junior high school	Han	ChronicHeartFailure (HCF)
P11	Male	64	married	Primary school	Han	Sick Sinus Syndrome(SSS)
P12	Male	66	married	Specialty	Han	Fibrillation
P13	Male	53	married	Primary school	Han	Syncope
P14	Female	88	married	Junior high school	Han	SSS
P15	Female	60	married	Primary school	Han	DCM
P16	Male	19	single	Senior high school	Han	Atrioventricular Block(AVB)
P17	Male	67	married	Junior high school	Han	coronary heart disease(CHD)
P18	Female	71	married	Primary school	Han	Bradycardia

**Table 2:** HCP’ characteristics (*N* = 20)

Role/Position	N(%)
physician	13(65%)
nurse	7(35%)
**Gender**	
Male	11(55%)
Female	9(45%)
**Age**	
20–25	2(10%)
26–30	5(25%)
31–35	10(50%)
36–40	3(15%)

### Themes

The in-depth interviews revealed four themes: Safety and success is priority; Humanistic Caring is a must yet be lacking; Paradox of surgery information given; Ways to improve surgery experiences in the operation.

#### Theme 1: safety and success is priority

For most patient participants, their desire was that the surgery could be completed successfully and they tried to play a “good patient” role. They stated that the guidance of HCPs should be fully followed and they must obey the doctors’ orders until the operation was completed. If they had discomfort in the surgery, they chose to endure suffering for not troubling HCPs.

They said that I can’t move,in my opinion, the doctors are always right, just obey them and do it…. Although my feet was cold, I didn’t say anything, only persist, persistence is victory… P6.

Some patient participants pointed out that they wanted to talk to HCPs about the surgery. However, they were also worried that their words and behaviors would interfere with HCPs’ work and increase the duration of surgery, which might hamper the normal procedure of the operation.

About the placement of guide wires and pacemakers, I want the medical workers to tell me. Otherwise, I didn’t know what happened in the surgery…but I wondered if I would interrupt their work, I think it may be better that I just stay silent. P10.

AllHCP participants were concerned about the safety and success of surgery, such as shorter surgery duration, efficiency of surgery, and postoperative complications. To reach this goal, patients should cooperate with HCPs and follow their orders.

The surgery result is the most important thing, instead of the process. To ensure the safety of operation, sometimes we have to sacrifice patients’ comfort. HCP19.

#### Theme 2: humanistic caring is a must yet be lacking

Patient participants acknowledged that they endured pain, anxiety, tension, etc. during the surgery. They said that they were alone in the operation room, and HCPs were concerned about the pacemaker, the vessel, the thresholds of parameter instead of patients. Whereas, what they wanted was accompany and being with them, which was a way of showing caring for patients.

This was my first surgery, I didn’t know what would happen when I was lying alone in the surgery bed, nobody talked to me, I felt nervous and fearful. After a long time, I felt better than before when the doctor came in. P9.

Some patient participants stated they hoped HCPs could communicate with them, no matter what the topic is, which could help them relaxed and provided a support and caring atmosphere for them.

I just want someone to talk to me, really, otherwise I would feel lonely that I couldn’t stay here. Just talk to me, it doesn’t matter what to say, chatting is ok. P3.

Some patient participants reported that they felt discomfort during the procedure, such as cold, pain, or breathless during the operation. They recalled that HCPs have taken some measures to alleviate their discomfort but they turned to focus on the operation quickly, and it seemed didn’t work. They had to endure the suffering and expected the surgery to be ended as soon as possible.

I thought the operation room should be warm. I was cold. After they noticed it, they adjusted the temperature and added sterile cloth, but I still felt so cold, especially my feet. HCPs were busy with the operation and nobody cared about my cold feet any more. The operation duration was so long. P6.

Most HCP participants mentioned that except surgery, actions such as communicating with patients to appease the negative emotion, paying attention to their comfort timely, and building trust and friendly relationship with patients were highlighted. They reflected that only completing the surgery while ignoring patient’s feelings would do harm to patients.

Most CIED surgery used local anesthesia,the patient was awake throughout the whole process. Therefore, care is more important during the surgery. We should do something to alleviate patients’ anxiety and tension to improve their experience. HCP19.

Some HCP participants stated that although humanistic caring is a necessity in the operation for its key role in today’s health care system, caring is still wanted in the surgery due to different reasons such as personnel shortage and lack of competency,

Care is an essential role in the development of medicine…However, we don’t have enough staff…. Now we have tried our best to finish surgery safely and efficiently. Besides, some staff are lacking competence to provide caring. HCP20.

#### Theme 3: Paradox of surgery information giving

Some patient participants pointed out that they were more curious about the procedure during the operation, they looked forward that HCPs could tell them the surgery information. Sometimes, they also took initiative in inquiring information.

I wanted to see what the pacemaker looked like, how the doctor performed the operation, and how long the operation took, etc. I’d like to know all of these. P9.

However, some patient participants stated that the information concerning surgery, such as operation processes and the size of the pacemaker, etc., would make them fearful and anxious.

I would be more nervous if they told me about the procedure of the operation P14.

Some HCP participants considered that information such as telling patients that the surgery was about to end, and brief introduction of operation information would benefit patients.

We often release a signal for them that the operation is almost over, so that they would not be too anxious, then it would be much easier for them. HCP18.

Patients may feel anxious for being alone and knowing nothing about what is going to happen around themselves… Our brief introduction about the operation will help.MP4.

Some HCP participants said that in order to prevent patients from focusing on surgical operations, which might make them nervous, it would be better to talk to patients with some other topics instead of surgery to create a relaxing atmosphere.

What we usually talk to patients is topics having nothing to do with the surgery, such as their family life, personal interest, etc., which may disperse their attention and help them relaxed. HCP7.

#### Theme 4: ways to improve surgery experiences in the operation

Some patient participants said that they held the belief that HCPs could handle everything and there was no need to worry about in the operation. Wait and rest is ok. Even if emergency occurred, that was their bad fortune and had nothing to do with HCPs.

I closed my eyes and had nothing to think, I didn’t feel nervous, just waiting for the surgery to be ended. P11.

There was a problem with the line, it’s not their fault, it is my bad luck…. P6.

Other patient participants usually thought of some wonderful things, such as the improved quality of life after a successful operation.

When I was nervous, I often focused on some wonderful things, such as, everything will be fine after the operation…. then I’m relaxed. P1.

Some HCP participants stated that there were some methods to improve patient’s experience, such as giving surgery information to patients ahead to help them prepare for the operation, and inspire patients to persist and complete the operation during the surgery.

We usually make patients understand the general information of the entire surgery through the preoperative conversation, including the surgery procedure, risks that may occur during the operation, etc. We will explain that in detail, and patients are fully prepared. HCP15.

We would comfort the patient that this surgery is just a minor operation and HCPs have extensive experience in surgery, encouraging them to be brave and persisting in until the surgery is completed. HCP7.

## Discussion

This qualitative study captured the perception of patients and HCPs on care experience of CIED surgery. The results showed that safety and success is priority and it should take precedence over anything, which is in accordance with the perceptual adjustment level theory by Matiti et al [35]. According to this theory, patients realize that being hospitalized with some suffering and loss of dignity is a worthwhile price to pay for the sake of safety, which is regarded as a ‘necessary submission’ [35], namely, patients need to subject to the hospital system, being told what to do and being dependent on health staff, and thereby losing their identity [36]. Furthermore, in Chinese culture, pain as a “trial” or “sacrifice’’ is profoundly meaningful. Therefore, when a person suffers with pain, he or she would rather endure it until the pain becomes unbearable [37]. As a result, patients who receive CIED surgery tend rationalize their positions such as pain in the operation, and they would rather accept the suffering and not report it to HCPs, because they know it is temporary and success of the surgery is priority [38]. Safety and success is also considered as the key issue by HCPs. A survey of 17 clinicians involved in CIED implantation showed that safety and success of the procedure are superior to patient comfort [14]. As healthcare institutions aim to offer high-quality care and the patient safety have become a major concern for healthcare facilities, many HCPs are aware of the effect of patient safety on patient outcomes [39, 40]. A large retrospective review reported that 66% of all adverse events(AEs) were related to surgery [41]. Consequently, every year, at least seven million patients suffer from surgical complications, including at least one million who die during or immediately after surgery [42]. Hence, surgical team are more focused on patient safety and have taken measures to reduce the rate of AEs, thus improving patient outcomes [43].

Under a profoundly stressful circumstance, patients often need more attention and support from HCPs, which suggests the importance of providing humanistic care [20, 44]. The necessity of humanistic caring was stressed by patients and HCPs as well in the study, which is in accordance with Chinese culture. Humanity, which is also known as benevolence, is an attitude which is considered to be the greatest of all virtues and at the roots of Chinese culture. One of the highest compliments being paid to a Chinese person is to say that he/she has the aura of a benevolent person [45]. Humanistic care refers to listening to the needs and desires of patients, understanding patients’ emotions and respecting their life values, which can help patients reach a higher level of physical, psychological, social and spiritual well-being [46, 47]. In the delivery of health care, especially with the development of patient-centered approach, there is a consensus on the importance of humanistic care in clinical practice [48]. Whittle et al [49] suggested that it is useful to decrease pain and discomfort during awake brain tumour surgery by providing a comfortable operating table, a dedicated person for patients communication, and keeping them warm. Willem et al [50] reported that patients undergoing awake craniotomy believed that humanistic care, such as positive interactions and support from HCPs, is important to reduce their fear and uncertainty. Our study results showed that HCPs realized the humanistic care was essential in the operations, but it is poorly implemented. The reason might be that nursing practice is driven by a complex system of humanistic dimension (including educational, social/cultural and spiritual), but is constrained and influenced by bureaucratic dimension (including technological, economic, legal and political),which is emphasized in the theory of Bureaucratic Caring [51, 52]. In the operation room, HCPs are under pressure to perform their work with maximal efficiency in a minimal amount of time, therefore, they often pay more attention to enhance care of technological dimension [53]. The humanistic dimension of caring might be neglected due to the fact that economic factors such as too much workload and shortage of staffing negatively influence direct care time [42, 54, 55]. Therefore, there is a need to focus on the interplay between bureaucratic and humanistic dimension to provide high quality care in operation setting.

A number of studies have reported that providing surgical information during the operation reduced patients’ anxiety and satisfy their caring needs [56, 57]. However, our findings indicate that surgical information was a burden for some patients undergoing CIED surgery such as increasing their intraoperative anxiety, which might be explained by the ‘Blunting Hypothesis’ proposed by Miller [58]. This hypothesis categorizes individuals into two different information styles (monitors, monitoring information-seeking styles or blunters, blunting information-seeking styles) in seeking, encoding, processing and managing threatening cases, such as CIED surgery [16, 58, 59]. During the CIED surgery, monitors typically seek threat-relevant information to reduce their uncertainty and promote feelings of reassurance [60]. In contrast, blunters prefer less information and their anxiety may be increased when information is delivered too much [61]. Hence, it is not surprising that the conflicting results emerged about patient’s response to CIED surgery information. It has been demonstrated that patients have better outcomes psychologically, behaviorally and physiologically when the amount of information received is consistent with the patients’ information-seeking styles [62]. In addition, patient involvement in clinical decision making has been increasingly advocated, and giving information to patients as a foundation for their involvement is valued by HCPs [63]. Kim et al [56] pointed that providing surgical information gave the patient the opportunity to manage their fears, increased patients participation, and resulted in increased well-being. Whereas, it is also evidenced by other studies that surgical information given in the operation might increase patients’ anxiety and HCPs may choose not to tell patients information about the procedure [64, 65], which is also demonstrated in our study. Besides, Anna et al [66] reported that talking to patients with some topics that had nothing to do with surgery is a way to distract patients’ attention and reduce their anxiety. Therefore, it is important for HCPs, based on the patient’s information-seeking styles, to develop an appropriate information support manner in the surgery.

In our analyses, there are four main approaches to improving patients’ intraoperative care experience. Patients may trust and rely on HCPs during the operation, and believe that HCPs will take care of everything. This finding is similar to those of Emel et al [10] who noted that patients release all control and responsibility to the HCPs when they are placed on the operating table for a surgical intervention. Eyi et al [67] also stated that it is vital to trust HCPs during the surgery. According to attachment theory, when individuals feel vulnerable in the face of major threats they seek attachment figures to help them feel safe [68]. In operation settings, HCPs are often in the position of an attachment figure because the patients view their providers as an “expert” with the skills to extend the quality and quantity of their life [69]. In China, individuals’ ways of living and thinking about health are influenced by several main Chinese philosophies and religions [16]. Taoism emphasizes harmony with nature, and conformity with nature is a process of knowing nature, trying to modify oneself to best fit nature, namely, as long as we try our best and going with the flow, the outcome is accepted peacefully [70, 71]. Therefore, patients often chose to make the best effort to co-operate and believe in HCPs during the operation. Our study results showed that patients adopted an optimistic psychological state to enhance intraoperative experience and looked forward to positive events after the surgery. This positive psychological assets is consistent with the idea proposed by Confucianism and Taoism that optimizing will reduce suffering and improve well-being [71, 72].

Preoperative information support was cited as an approach to improve patients’ care experience for HCPs, which has been demonstrated in other researches [73]. A study showed that good patient preoperative counseling allays patients’ anxiety and facilitates successful surgery under local anesthesia [74]. Similarly, Emel et al [10] also emphasized the importance of preoperative information in reducing patients’ anxiety and fear levels under spinal anesthesia. As an important component of psychological preparation, preoperative information support is effective for patients with conscious state to reduce anxiety and pain [75, 76]. Stefan et al [77] suggested that we should take more time to provide information and support before operation in order to improve patients’ positive experience and surgical outcomes. Our study found that inspiring patients with prior patients’ successful experiences by HCPs acts as a way of therapeutic suggestion, which can reduce side effect and increase comfort of patients during CIED, and it is also reflected by other studies in various medical procedures, surgical procedures and chemotherapy [78]. Christine et al [79] found that therapeutic suggestions can diminish patients’ pain, anxiety and procedure time during radiological procedures. In addition, as a simple communicational technique, the method of therapeutic suggestion can easily be incorporated into everyday work in a clinical environment and can be readily learned by HCPs [78].

### Strengths and limitations

The perceptions of patients undergoing CIED surgery regarding intraoperative care in the Chinese context are rarely explored in the literature. This study also presents HCPs’ perceptions to better understand the experience of intraoperative care, which brings new and interesting information for further research to develop intraoperative care programs that can benefit patients. One of the limitations of this study was that the participants were from only one region of China, which limits the generalisability of the results, and caution should be taken when reading the results. In addition, HCPs included in this study only involve physicians and nurses, and professions such as technicians are lacking, which can be considered for future studies. Furthermore, this study was conducted in Chinese environment, and different cultural environments may lead to diverse experiences and perspectives. Further studies can be conducted in many other hospitals and among different ethnic groups to enrich the results. More researches are necessary to explore CIED patients’ experience during the surgery based on multiple stakeholders’ perspectives, which can contribute to developing a comprehensive and systematic intraoperative care programme.

## Conclusion

Based on patients’ and HCPs’ perspectives, patients who underwent CIED surgery face psychological and physical stresses, which interfere with their comfort and well-being. This study demonstrates the complexity and challenges of providing intraoperative care for HCPs. To improve care experience during the surgery, HCPs should pay attention to patients’ safety, besides, information support should consider patients’ information-seeking styles and personal needs. As an important part of intraoperative care, the factors that affecting humanistic care in clinical practice should be valued by healthcare facilities and measures should be taken to improve patients’ experience, thus achieving patient-centered care [80]. In addition, the approaches presented in this study are useful to improve intraoperative experience for patients and HCPs. Trusting HCPs and going with the flow, maintaining positive psychological state suggest the importance of building rapport between HCPs and patients, besides, culture variant as a vital factor in influencing individuals’ health beliefs and behaviors should be highlighted. Furthermore, preoperative information support and therapeutic suggestion are effective ways that can be easily implemented by HCPs.

### Electronic supplementary material

Below is the link to the electronic supplementary material.

**Supplementary Material 1:** Interview guide

## Data Availability

The data that support the findings of this study are available on request from the corresponding author. The data are not publicly available due to privacy or ethical restrictions.

## References

[1] Mian M, Khan HR (2023). Ultrasound utilization for implantation of cardiac implantable electronic devices. Wien Klin Wochenschr.

[2] Steffen MM, Osborn JS, Cutler MJ (2019). Cardiac Implantable Electronic device therapy. Med Clin North Am.

[3] Khalighi K, Aung TT, Elmi F (2014). The role of Prophylaxis Topical antibiotics in Cardiac device implantation: CARDIAC DEVICE IMPLANTATION. Pacing Clin Electrophysiol.

[4] Okajima T, Inden Y, Yanagisawa S, Suga K, Shimojo M, Nakagomi T (2022). Impact of synchronized left ventricular pacing rate on risk for ventricular tachyarrhythmias after cardiac resynchronization therapy in patients with heart failure. J Interv Card Electrophysiol.

[5] Helmark C, Egholm CL, Rottmann N, Skovbakke SJ, Andersen CM, Johansen JB (2023). A web-based intervention for patients with an implantable cardioverter defibrillator– A qualitative study of nurses’ experiences (data from the ACQUIRE-ICD study). PEC Innov.

[6] E K, Ra HSJLTR. J. Feasibility and safety of using local anaesthesia with conscious sedation during complex cardiac implantable electronic device procedures. Sci Rep. 2018;8. 10.1038/s41598-018-25457-x PMC5940700 29740019

[7] Kodvavi MS, Asghar MA, Ghaffar RA, Nadeem I, Bhimani S, Kumari V (2023). Effectiveness of virtual reality in managing pain and anxiety in adults during periprocedural period: a systematic review and meta-analysis. Langenbecks Arch Surg.

[8] Aj G, Jd P, Ja EL, Dr O, Rt F. H, Trends in permanent pacemaker implantation in the United States from 1993 to 2009: increasing complexity of patients and procedures. J Am Coll Cardiol. 2012;60. 10.1016/j.jacc.2012.07.017 22999727

[9] Marrocco-Trischitta MM, Tiezzi A, Svampa MG, Bandiera G, Camilli S, Stillo F (2004). Perioperative stress response to carotid endarterectomy: the impact of anesthetic modality. J Vasc Surg.

[10] Yilmaz E, Toğaç HK, Çetinkaya A, Toğaç S (2020). A qualitative study of the operating room experience of patients who underwent surgery under spinal anesthesia: it was like an adventure. Nurs Health Sci.

[11] Sigdel DrS. Perioperative anxiety: a short review. Glob AnesthPerioper Med. 2015;1.

[12] Bheemanna N, Channaiah SD, Gowda PKV, Shanmugham V, Chanappa N (2017). Fears and perceptions associated with regional anesthesia: a study from a tertiary care hospital in South India. Anesth Essays Res.

[13] van der Acj L, Mt R, Am H, Vp van SE, Nm H. B, The link between cardiac status and depression and anxiety in implantable cardioverter defibrillator patients: design and first results of the PSYCHE-ICD study. J Psychosom Res. 2023;167. 10.1016/j.jpsychores.2023.111182 36801661

[14] Pain during cardiac implantable electronic device implantation (2021). Br J Cardiol.

[15] Brandt T, Huppert D (2021). Brain beats heart: a cross-cultural reflection. Brain.

[16] Zhuo Q, Liang H, Bai Y, Hu Q, Hanum AL, Yang M (2021). Perceptions of patients undergoing percutaneous coronary intervention on pre-operative education in China: a qualitative study. Health Expect.

[17] Rufai SR, Mitchell BG, Farmer TD, Lash SC (2015). Reducing anxiety during conscious surgery– a patient survey. Int J Surg.

[18] Im G. F T. Effects of massage and acupressure on relieving labor pain, reducing labor time, and increasing delivery satisfaction. J Nurs Research: JNR. 2020;28. 10.1097/jnr.0000000000000344 31524645

[19] Haugen AS, Eide GE, Olsen MV, Haukeland B, Remme ÅR, Wahl AK (2009). Anxiety in the operating theatre: a study of frequency and environmental impact in patients having local, plexus or regional anaesthesia: *anxiety in the operating theatre*. J Clin Nurs.

[20] Turesson C, Kvist J, Krevers B (2019). Patients’ needs during a surgical intervention process for Dupuytren’s disease. Disabil Rehabil.

[21] Bergman M, Stenudd M, Engström Å (2012). The experience of being awake during orthopaedic surgery under regional anaesthesia. Int J Orthop Trauma Nurs.

[22] Js M, Ks C. The effects of handholding on anxiety in cataract surgery patients under local anaesthesia. J Adv Nurs. 2001;35. 10.1046/j.1365-2648.2001.01855.x 11489026

[23] Merakou K, Varouxi G, Barbouni A, Antoniadou E, Karageorgos G, Theodoridis D et al. Blood pressure and heart rate alterations through music in patients undergoing cataract surgery in Greece. Ophthalmol Eye Dis. 2015;7:OED.S20960. 10.4137/OED.S20960 PMC4467656 26106264

[24] Ij EH, Js B, Ms B, Ps M, Mc H. S, Virtual reality improves the patient experience during wide-awake local anesthesia no tourniquet hand surgery: a single-blind, randomized, prospective study. Plast Reconstr Surg. 2019;144. 10.1097/PRS.0000000000005831 31348351

[25] Hejrati N, Spieler D, Samuel R, Regli L, Weyerbrock A, Surbeck W (2019). Conscious experience and psychological consequences of Awake Craniotomy. World Neurosurg.

[26] Pain after pacemaker/ICD implants (2022). Br J Cardiol.

[27] Areti Stavropoulou D, Vlamakis E, Kaba I, Kalemikerakis M, Polikandrioti G, Fasoi (2021). Living with a Stoma: exploring the lived experience of patients with Permanent Colostomy. IJERPH.

[28] Neubauer BE, Witkop CT, Varpio L (2019). How phenomenology can help us learn from the experiences of others. Perspect Med Educ.

[29] Wang P, Li Y-R, Ge H, Liu J-Y, Li S-W (2023). Experience in developing innovative practical ability for master of nursing specialist degree program in China: a qualitative descriptive study of postgraduates. Nurse Educ Today.

[30] Tong A, Sainsbury P, Craig J (2007). Consolidated criteria for reporting qualitative research (COREQ): a 32-item checklist for interviews and focus groups. Int J Qual Health Care.

[31] Chow C-S (2005). Cultural diversity and tourism development in Yunnan province, China. Geography.

[32] Wang Y (2021). On the necessity and strategies of promoting the influence of yunnan minority sports culture under the background of the belt and road initiative initiative. IOP Conf Ser: Earth Environ Sci.

[33] Wang X, Liu L (2007). Cultural barriers to the use of western project management in Chinese enterprises: some empirical evidence from Yunnan province. Project Manage J.

[34] Braun V, Clarke V (2006). Using thematic analysis in psychology. Qualitative Res Psychol.

[35] Matiti MR, Trorey G (2004). Perceptual adjustment levels: patients’ perception of their dignity in the hospital setting. Int J Nurs Stud.

[36] Nilsson G, Larsson S, Johnsson F, Saveman B-I (2002). Patients’ experiences of illness, operation and outcome with reference to gastro-oesophageal reflux disease. J Adv Nurs.

[37] Lm C, C M. M D, S P. Concepts within the Chinese culture that influence the cancer pain experience. Cancer Nurs. 2008;31. 10.1097/01.NCC.0000305702.07035.4d 18490884

[38] Mauleon AL, Palo-Bengtsson L, Ekman S-L (2007). Patients experiencing local anaesthesia and hip surgery. J Clin Nurs.

[39] Lee SE, Scott LD, Dahinten VS, Vincent C, Lopez KD, Park CG (2019). Safety Culture, Patient Safety, and Quality of Care outcomes: a Literature Review. West J Nurs Res.

[40] AL-Mugheed K, Bayraktar N, Al-Bsheish M, AlSyouf A, Jarrar M, AlBaker W (2022). Patient safety attitudes among doctors and nurses: associations with workload, adverse events, experience. Healthcare.

[41] Etchells E, O’Neill C, Bernstein M (2003). Patient safety in surgery: Error Detection and Prevention. World J Surg.

[42] Aouicha W, Tlili MA, Sahli J, Mtiraoui A, Ajmi T, Said Latiri H (2022). Patient safety culture as perceived by operating room professionals: a mixed-methods study. BMC Health Serv Res.

[43] The Lancet (2016). Patient safety is not a luxury. The Lancet.

[44] Sundqvist A-S, Holmefur M, Nilsson U, Anderzén-Carlsson A (2016). Perioperative Patient Advocacy: an integrative review. J PeriAnesthesia Nurs.

[45] Sun CT-L. Themes in Chinese psychology. Published by Cengage Learning Asia and marketed by. World Scientific Publishing Co.; 2008.

[46] Wang Y, Zhang Y, Liu M, Zhou L, Zhang J, Tao H (2020). Research on the formation of humanistic care ability in nursing students: a structural equation approach. Nurse Educ Today.

[47] Yanhua Zhang (2022). Application Research of Humanistic Care and situational integration in nursing of Schizophrenia in Recovery Period. Contrast Media Mol Imaging.

[48] FakhredinTaghinezhad E, Mohammadi M, Khademi (2022). AnoshirvanKazemnejad. Humanistic Care in nursing: Concept Analysis using Rodgers’ Evolutionary Approach. Iran J Nurs Midwifery Res.

[49] Ir W, Am SMHG. P, R T. Patient perceptions of awake brain tumour surgery. Acta Neurochir. 2005;147. 10.1007/s00701-004-0445-7 15627921

[50] Potters J-W, Klimek M (2015). Awake craniotomy: improving the patient’s experience. Curr Opin Anaesthesiol.

[51] Ray MA (1989). The theory of bureaucratic caring for nursing practice in the organizational culture. Nurs Adm Q.

[52] Ray MA, Turkel MC (2014). Caring as emancipatory nursing praxis: the theory of Relational Caring Complexity. Adv Nurs Sci.

[53] Ingvarsdottir E, Halldorsdottir S (2018). Enhancing patient safety in the operating theatre: from the perspective of experienced operating theatre nurses. Scand J Caring Sci.

[54] Manworren RCB (2022). Nurses’ management of children’s acute postoperative pain: a theory of bureaucratic caring deductive study. J Pediatr Nurs.

[55] Mohamadi Asl S, Khademi M, Mohammadi E (2022). The influential factors in humanistic critical care nursing. Nurs Ethics.

[56] Kim B-H, Kang H-Y, Choi E-Y (2015). Effects of handholding and providing information on anxiety in patients undergoing percutaneous vertebroplasty. J Clin Nurs.

[57] Chit Ying L, Levy V, Oi Shan C, Wing Hung T, Kit Wah W (2001). A qualitative study of the perceptions of Hong Kong Chinese women during caesarean section under regional anaesthesia. Midwifery.

[58] Miller SM, Levine S, Ursin H (1980). When is a little information a dangerous thing? Coping with stressful events by Monitoring Versus Blunting. Coping and Health.

[59] Miller SM (1995). Monitoring versus blunting styles of coping with cancer influence the information patients want and need about their disease. Implications for cancer screening and management. Cancer.

[60] Miller SM. Monitoring and blunting: validation of a questionnaire to assess styles of Information Seeking Under Threat. 10.1037//0022-3514.52.2.345 3559895

[61] Zhuo Q, Cui C, Liang H, Bai Y, Hu Q, Hanum AL (2021). Cross-cultural adaptation, validity and reliability of the Chinese Version of Miller behavioral style scale. Health Qual Life Outcomes.

[62] Kola S, Walsh JC, Hughes BM, Howard S (2013). Matching intra-procedural information with coping style reduces psychophysiological arousal in women undergoing colposcopy. J Behav Med.

[63] Robben S, Van Kempen J, Heinen M, Zuidema S, Olde Rikkert M, Schers H (2012). Preferences for receiving information among frail older adults and their informal caregivers: a qualitative study. Fam Pract.

[64] Lim L, Chow P, Wong C-Y, Chung A, Chan Y-H, Wong W-K (2011). Doctor–patient communication, knowledge, and question prompt lists in reducing preoperative anxiety– a randomized control study. Asian J Surg.

[65] Berger ZD, Boss EF, Beach MC (2017). Communication behaviors and patient autonomy in hospital care: a qualitative study. Patient Educ Couns.

[66] Abelsson A, Falk P, Sundberg B, Nygårdh A (2021). Empowerment in the perioperative dialog. Nurs Open.

[67] Eyi S, Kanan N, Akyolcu N, Akn ML, Acarolu R (2016). Evaluation of intraoperative nursing care by patient. TAF Prev Med Bull.

[68] Frederiksen HB, Kragstrup J, Dehlholm-Lambertsen B (2010). Attachment in the doctor–patient relationship in general practice: a qualitative study. Scand J Prim Health Care.

[69] Palmer Kelly E, Tsilimigras DI, Hyer JM, Pawlik TM (2019). Understanding the use of attachment theory applied to the patient-provider relationship in cancer care: recommendations for future research and clinical practice. Surg Oncol.

[70] Chen Y (2001). Chinese values, health and nursing. J Adv Nurs.

[71] Chen Y-LD. Conformity with nature: a theory of Chinese American Elders’ Health Promotion and Illness Prevention processes: advances in nursing science. 1996;19:17–26. 10.1097/00012272-199612000-00004 8939285

[72] Park N, Peterson C, Szvarca D, Vander Molen RJ, Kim ES, Collon K (2016). Positive Psychology and Physical Health: Research and Applications. Am J Lifestyle Med.

[73] Guo P, East L, Arthur A (2012). A preoperative education intervention to reduce anxiety and improve recovery among Chinese cardiac patients: a randomized controlled trial. Int J Nurs Stud.

[74] Mr M, La W. R H. Surgeon experience and patient comfort during clear corneal phacoemulsification under topical local anesthesia. J Cataract Refract Surg. 2002;28. 10.1016/s0886-3350(02)01369-x 12457673

[75] Guo P (2015). Preoperative education interventions to reduce anxiety and improve recovery among cardiac surgery patients: a review of randomised controlled trials. J Clin Nurs.

[76] Tomaszek L, Cepuch G, Fenikowski D (2019). Influence of preoperative information support on anxiety, pain and satisfaction with postoperative analgesia in children and adolescents after thoracic surgery: a randomized double blind study. Biomed Pap Med Fac Univ Palacky Olomouc Czech Repub.

[77] Salzmann S, Euteneuer F, Kampmann S, Rienmüller S, Rüsch D (2023). Preoperative anxiety and need for support– a qualitative analysis in 1000 patients. Patient Educ Couns.

[78] K Szilágyi A, Kekecs Z, Varga K (2018). Therapeutic suggestions with critically ill in palliative care. Ann Palliat Med.

[79] Cj S, K B MB, Ev L. Pain and anxiety during interventional radiologic procedures: Effect of patients’ state anxiety at baseline and modulation by nonpharmacologic analgesia adjuncts. J Vascular Interventional Radiology: JVIR. 2005;16. 10.1097/01.RVI.0000185418.82287.72 16371522

[80] Jardien-Baboo S, Van Rooyen D (R. M), Ricks EJ, Jordan PJ. Ten Ham‐Baloyi W. Integrative literature review of evidence‐based patient‐centred care guidelines. J Adv Nurs. 2021;77:2155–65. 10.1111/jan.14716 33314226

