# Supplementary material for: A qualitative study on patients’ and health care professionals’ perspectives regarding care delivered during CIED operation
Source: BMC Health Serv Res. 2024 Jan 15;24:73. doi: 10.1186/s12913-024-10546-7 (PMC10789075; doi:10.1186/s12913-024-10546-7)
Supplement: Supplementary file 1 — Supplementary Material 1: Interview guide [file 12913_2024_10546_MOESM1_ESM.docx]

**Interview guide**

**Patient participants**

1. What did you remember about the intraoperative experience? What you heard and observed?

2. How did you feel during the operation? Please describe your emotions that you can remember……and please describe your thoughts that you can remember......

3. During the operation, was there anything that particularly impressed you? How come?

4. What was your impression of the medical staff during the operation? In your opinion, what ought they to do?

5. Do you have any suggestions or information to share?

**Healthcare professional participants**

1. As a physician/nurse, what was your primary duty during the operation, and what would you do for the patients?

2. How do you understand the intraoperative care? How can the patients be best taken care of?

3. During the operation, was there anything that particularly impressed you? How come?

4. How did the patients feel during the operation? How else are you able to help them?

5. Do you have any suggestions or information to share?
